# The Royal Army Medical Corps

**Published:** 1914-09-05

**Authors:** 


### THE ROYAL ARMY MEDICAL CORPS.

A candidate for a commission in the Royal Army Medical Corps must be 21 years and not over 28 years of age at the date of the commencement of the Entrance examination. He must be registered under the Medical Acts in force in the United Kingdom. Before being permitted to proceed to the Entrance examination, the candidate is required to attend at the War Office about two days before the commencement for the purpose of being interviewed and undergoing physical examination. The Entrance examination is held twice a year in London—January and July—a fee of £1 being charged for admission thereto. It lasts about three days. The examination is of a clinical, written, practical, and oral character. It consists of: A commentary on a case in medicine; an essay in surgery; examination and report on a clinical medical case; examination and report on clinical surgical cases; orals in clinical medicine, clinical surgery and in medical and in surgical pathology; also surgical operations and bandaging (including surgical instruments and appliances). A candidate who is successful at the Entrance examination is appointed a lieutenant (on probation) and is then required to attend at the Royal Army Medical College, London, and afterwards at the Royal Army Medical Corps School of Instruction, Aldershot, for the purpose of qualifying in the following subjects before his commission can be confirmed:—Royal Army Medical College: Military surgery, tropical medicine, hygiene, pathology, and organisation of military hospitals, and principles governing medical charge of troops. Royal Army Medical Corps School of Instruction: Regimental duties, drill and field training.

Further particulars relating to marks given for service in the O.T.C. or T.F., etc., and regarding the holding of civil appointments after having passed the Entrance examination are contained in "Regulations for admission to the R.A.M.C." which can be obtained on application to the Secretary, War Office.

